# Elucidating the complex organization of neural micro-domains in the locust *Schistocerca gregaria* using dMRI

**DOI:** 10.1038/s41598-021-82187-3

**Published:** 2021-02-09

**Authors:** Syed Salman Shahid, Christian M. Kerskens, Malcolm Burrows, Alice G. Witney

**Affiliations:** 1 Department of Radiology and Imaging Sciences, Indiana University School of Medicine, Indianapolis, IN USA; 2 Trinity College Institute of Neuroscience, Trinity Centre for Biomedical Engineering, School of Medicine, Trinity College Dublin, Dublin 2, Ireland; 3 Department of Zoology, University of Cambridge, Cambridge, UK; 4 Department of Physiology, School of Medicine, Trinity Biomedical Sciences Institute, Trinity Centre for Biomedical Engineering, Trinity College Institute of Neuroscience, Trinity College Dublin, Dublin 2, Ireland

**Keywords:** Biological techniques, Biophysics, Neuroscience, Physiology, Zoology, Physics

## Abstract

To understand brain function it is necessary to characterize both the underlying structural connectivity between neurons and the physiological integrity of these connections. Previous research exploring insect brain connectivity has typically used electron microscopy techniques, but this methodology cannot be applied to living animals and so cannot be used to understand dynamic physiological processes. The relatively large brain of the desert locust, *Schistercera gregaria* (Forksȧl) is ideal for exploring a novel methodology; micro diffusion magnetic resonance imaging (micro-dMRI) for the characterization of neuronal connectivity in an insect brain. The diffusion-weighted imaging (DWI) data were acquired on a preclinical system using a customised multi-shell diffusion MRI scheme optimized to image the locust brain. Endogenous imaging contrasts from the averaged DWIs and Diffusion Kurtosis Imaging (DKI) scheme were applied to classify various anatomical features and diffusion patterns in neuropils, respectively. The application of micro-dMRI modelling to the locust brain provides a novel means of identifying anatomical regions and inferring connectivity of large tracts in an insect brain. Furthermore, quantitative imaging indices derived from the kurtosis model that include fractional anisotropy (FA), mean diffusivity (MD) and kurtosis anisotropy (KA) can be extracted. These metrics could, in future, be used to quantify longitudinal structural changes in the nervous system of the locust brain that occur due to environmental stressors or ageing.

## Introduction

A key challenge of neuroscience is understanding the emergence of behaviour from neuronal activity. The idea that neuronal circuit formation shares common design principles across different species to generate behaviourally equivalent outputs has had a long history since the pioneering work of Ramon y Cajal and his early observations of the similarity in organization between insect and human visual processing^1^. Interestingly, regardless of the number of neurons that comprise the brain of an animal and morphological differences, evidence suggests that the basic principles underlying neuronal connectivity are similar^2,3^. The emergent field of connectomics develops from the premise that the understanding of complex brain function must be related to its structural underpinnings^4^. The construction of maps of structural connectivity in the nervous system has subsequently become a widespread aim of the neuroscience community^5^. Together this has led to the emerging field of connectomics which, dependent on animal, has explored structural connectivity at a synaptic resolution or the ‘microscale connectome’ or characterized tract connectivity between brain regions at the ‘macroscale connectome’^5^. For the field of connectomics to develop and further reveal how alterations in structure impact on behaviour it has been argued more comparative studies should be conducted with the same technique applied across diverse species, as well as the application of multiple imaging techniques to the same species^5^ so that the benefits of each methodology can provide a more comprehensive understanding of the properties of the neuronal network and its impact on behaviour.

Contrasting techniques have been utilized in invertebrate compared with mammalian connectomics research^5^. Invertebrate connectomics can achieve synaptic level resolution of connectivity in the brain^6^ through the use of electron microscopy^7,8^. The complete connectome of *C. elegans*^9^ and *Drosophila*^8^ can been constructed through semi-automatic processing of high resolution image stacks to produce reconstructed images^10^. The reconstruction of electron microscopy images remains a time realistic approach for these animals, given their simpler nervous system, and one that has begun to characterize relationships between neuronal architecture and behaviour. However, there are limitations to this methodology, and the approach is difficult to apply to animals with more complex nervous systems as reconstructions of electron microscopy images remain time consuming^11^. Recent technological developments utilizing volumetric fluorescent microscopy has provided increased processing speed combined with high resolution and so will enable connectomes of other species with more complex nervous systems^12^. However, typical microscopy techniques currently used for invertebrate connectomics tend to necessitate the animal being dead and consequently present a static image of neuronal connectivity^13^ and so cannot be the sole technique for understanding how neuronal network activity relates to behaviour. Therefore within invertebrates, other techniques that can image network level neuronal activity in live behaving animals have been additionally explored including calcium and voltage imaging to observe functional connectivity^14^. Further, micro computed tomography (µCT) has been applied as a technique that can provide anatomical details at high resolution non-invasively^15^. However the high levels of radiation associated with µCT mean this methodology does not have potential for longitudinal imaging and this method does not provide information about functional connectivity in the brain. In contrast to invertebrate connectomics mammalian connectomics tends to rely on ‘macroscale’ resolution with instead the focus on inferred tract connectivity between brain regions and global network level organization. However, mesoscale resolution has been achieved through the additional use of anterograde or retrograde tracers. In mammalian research, these structural connectivity studies have been facilitated by the advent of diffusion magnetic resonance imaging (dMRI)^16^. The key benefit of MRI is that the technique enables live imaging of structural connectivity in an intact animal. Arguably, longitudinal studies of neuronal networks over an animal’s lifespan are needed to truly understand the link between neuronal networks and adaptable behaviours. Furthermore, uniquely, recent developments in dMRI are thought to able to quantify micro-structural changes that occur that may reflect damage, ageing or adaptive plasticity in the nervous system^17,18^. That is even changes that occur at axons and cell membranes may be reflected in quantitative metrics derived from dMRI and the technique has high sensitivity to alterations in tissue micro-architecture^19^. Therefore the technique provides a methodology that could enable mechanistic questions to be addressed with regard to linking how alterations in neuronal functioning that occur due to environmental factors or disease processes might be related to biological changes that occur in fibre tracts during development, ageing or injury. While dMRI has already been widely applied to human clinical research^17,20^, micro-scale dMRI is a rapidly developing area with potentially wider applications in imaging biological tissue.

The desert locust *Schisterocera gregaria* is a particularly important insect to use as a proof of concept to explore the application of dMRI for the characterization of a microscale diffusion environment in invertebrates. The locust has a large brain for an insect but furthermore is known to demonstrate an extreme form of environmentally driven polyphenism^21^. That is, dependent on sensory input, the animal’s genome can give rise to two different forms of animal; the non-migratory solitarious phase and the migratory gregarious phase. These two phases are known to be reversible and the animal exhibits extensive neural plasticity corresponding to behavioural changes^22–24^. Given the known neural plasticity in the locust, dMRI could prove to be particularly important additional technique for understanding and quantifying alterations of brain volumes and also in the micro-architecture of tracts in the locust brain^22^.

Magnetic resonance imaging and the modelling of the diffusion weighted imaging (DWI) via diffusion tensor imaging (DTI) is a very powerful and versatile method which can be made sensitive to underlying structure varying from non-biological structures through to biological tissues^25–29^. The method has become particularly widespread in mammalian research as it provides non-invasive and non-destructive imaging^30^. The body of all animals, including insects, has a high water content, and in a biological tissue water molecules are in a constant state of random motion. dMRI utilizes the principle that differences in water diffusion in tissues can be used to indirectly infer underlying anatomy via the direction of preferential diffusion and from this provide unique information on their microstructural architecture^31^. dMRI provides diffusion estimates for each voxel in a series of DW images. DTI and higher order diffusion schemes can then describe the estimated water self-diffusion in different dimensions by a diffusion tensor or more advanced representation schemes^32^. This tensor mathematically represents how the composite architecture of the body provides structural barriers differentially restricting the directions of water diffusion. The directionality of water diffusion that emerges due to these biological barriers in the body is known as anisotropic or directional diffusion, in contrast to isotropic diffusion where there is no directionality. In the brain the biological barriers to water diffusion include axons, cellular membranes and proteins. From this mathematical representation DTI indirectly allows the visualization of fibre pathways and connectivity. Importantly, DTI does not directly visualise axonal connections but is a statistical inference of structural connectivity^33,34^. However DTI is limited by assumptions intrinsic to it regarding the Gaussian distribution of water self-diffusion within the body, a simplification that will reduce the accuracy of the detail that the method can provide about the structures. This detail has nonetheless been sufficient for many applications. Diffusion weighted imaging has previously be applied to image a fixed insect brain^35^. However, in that study even though the spatial resolution of their DWI was 10 µm isotropic, the lack of angular resolution due to considering only a single diffusion direction hindered the possibility of visualization of internal structures.

Diffusion Kurtosis Imaging (DKI) is a newer method that adds additional dimensionality to the model of diffusion and attempts to capture the non-Gaussian distribution of diffusion to give better insights into differences in the barrier for molecular diffusion and from this provide a more accurate inference of the structure of the underlying tissue^20^. DTI or DKI has not previously been implemented on either live or ex-vivo insect brains but the application of dMRI and DWI scans provides the advantage of providing a fast means of visualising the brain and inferring neuronal tract connectivity non-invasively and non-destructively^36,37^. Further the application of dMRI enables the novel opportunity for quantification of indices that are thought to be able to reflect micro-structural alterations that are occurring in axonal tracts due to disease, injury or plasticity. For the locust this is potentially interesting due to the known neural plasticity associated with phase change^22^. Further due to the known commonalities at the cellular and molecular level between insects and vertebrates, the use of these quantitative measures in an insect model in combination with genetic tools^38^ has potential in the future to provide insights into axonal biology in vivo.

## Materials and methods

### Sample preparation

Three female 5th instar desert locusts, *Schistercera gregaria* (Forksȧl) (Blades Biological, Kent, UK) were decapitated by cutting rostral to the pronotum. Each locust head was then put in standard locust physiological saline^39^ before stabilizing in a 10 mm falcon tube, with the head immobilized by plastic holders. The antennae were removed from the locust head to facilitate good stabilization of the head within the falcon tube.

One female adult locust was used for the live scan. Prior to the live scan, the locust was cold-anesthetised in a fridge at around 4 °C for five minutes. The locust was wrapped in a dry, clean cloth from the pronotum down, leaving the head exposed. The fabric allowed respiration but restrained movement. The locust was then positioned on a purpose built plastic holder and taped to secure. To avoid motion artefacts resulting from head movements, a small piece of soft Styrofoam (~ 1 cm) was clamped between the end of the holder and the locusts’ head. Since the antennae would be difficult to stabilize during scanning they were removed.

### MR imaging

The dMRI acquisition protocol was optimised by the selection of diffusion parameters that enabled the scale of the diffusion attenuated signal to be made sensitive to the required dimensions^18^. This then allowed the modelling of the microscale diffusion environment of the locust brain.

The DWI data were acquired at the bore temperature on a horizontal bore 7 T Biospec micro-MRI system (Bruker, Etlingen Germany) equipped with shielded gradients (maximum gradient strength = 770 mT/m, rise time = 115 µs) and ^1^H mouse cryogenic surface coil (cryoProbe, Bruker Biospin). The acquisition was conducted in coronal plane (for the live locust, the acquisition was conducted in the axial plane) with the following acquisition parameters: 2D spin echo based diffusion sequence with unipolar diffusion sensitizing pulse field gradients placed symmetrically around the 180° RF pulse. TE/TR = 17.628/1000 ms; FOV = 7.5 × 10 mm; matrix size 96 × 128; slice thickness 0.781 mm; voxel size = 78.125 µm^3^. No inter slice spacing; number of slices = 35; no fat suppression; δ/Δ = 3.1/8.502 ms. The prescribed b-value = 800, 1800 and 2500 s/mm^2^, 6 directions for the first shell (b = 800 s/mm^2^), 8 directions for the second shell (b = 1800s/mm^2^) and 12 directions for the third shell (b = 2500 s/mm^2^). In total, there were 1 b0 image per shell and a total of 26 diffusion sensitizing gradient directions. The entire DWI volume was collected in a total scan time of ≈ 24 h.

### Diffusion MRI modelling

In a pulsed gradient spin-echo (PGSE) diffusion sequence^40^, if the diffusion-encoding gradient with amplitude *G* has infinitesimally short pulse duration *δ* compared to the diffusion time *Δ*, then the displacement of water molecules during this short diffusion gradient time can be ignored. Therefore, under the assumption of narrow pulse approximation (δ <  < Δ)^41^, the signal attenuation due to the diffusion-encoding gradient is given by:

1 $$\frac{S(q,\Delta )}{{S(0)}} = \int {P(R,\Delta )e^{2\pi iqR} dR}$$

where *q* = *γGδ/2π* is a wave vector^42^, *γ* is the gyromagnetic ratio, *R* is the dynamic displacement (displacement of spins during the allowed diffusion time *Δ)*, and *P(R)* is the ensemble average propagator (EAP) and it provides averaged estimate of the diffusion environment. The diffusion profile of a complex structure can be obtained using the inverse Fourier transform of the signal with respect to the wave vector^42^ and the excess kurtosis can be calculated using the following relation^43^:

2 $$K = \frac{{\mu_{4} }}{{\mu_{2}^{2} }} - 3 = \frac{{\kappa_{4} }}{{\kappa_{2}^{2} }}$$

where *µ*_*i*_ $= \smallint R^{n} P\left( R \right)dR,$ the cumulants (*κ*_*i*_) can be described in terms of moments of probability distribution (*µ*_*i*_). The first three cumulants (*κ*_*1-3*_*)* are equal to the first three moments (*µ*_*1*-3_). *µ*,* µ*_*2*_ and *µ*_*3*_ are the mean, variance and the skewness of the distribution, respectively. The fourth cumulant is related to the kurtosis as shown in Eq. (2). Diffusion Kurtosis can be derived from dMR signal by using the Fourier relationship between the attenuated signal and the EAP. The logarithm of the diffusion signal can be expanded as a summation of the cumulants *κ*_*n*_ of *P(R)*^43^:

3 $$\ln \frac{S(q)}{{S(0)}} = \sum\limits_{p = 1}^{\infty } {\kappa_{n} \frac{{\left( {2\pi iqR} \right)^{p} }}{p!}}$$

Under the assumption that diffusion is symmetrical (symmetric EAP), the phase of the attenuated signal can be considered zero, therefore, all odd order cumulants are null, i.e.,

4 $$\ln \frac{S(q)}{{S(0)}} = - \kappa_{2} \frac{{\left( {2\pi q} \right)^{2} }}{2} + \kappa_{4} \frac{{\left( {2\pi q} \right)^{4} }}{4!} + \kappa_{6} \frac{{\left( {2\pi q} \right)^{6} }}{6!} + ...$$

For isotropic Gaussian diffusion in time *Δ*, the diffusion coefficient can be expressed as: *D* = *κ*_*2*_*/(2Δ)*^36^, and by substituting it in Eq. (2), the fourth cumulant can be written as: *κ*_*4*_ = *4KD*^*2*^*Δ*^*2*^.

Under the assumption of PGSE, the diffusion weighting parameter ‘b-value’ is defined as:

5 $$b = \gamma^{2} G^{2} \delta^{2} \Delta$$

Using the relations of the second and fourth cumulants and Eq. (5), the signal attenuation can be approximated by the quadratic exponential kurtosis model after truncating Eq. (4) to the second term:

6 $$\ln \frac{S(b)}{{S(0)}} \approx - bD_{app} + \frac{1}{6}b^{2} D_{app}^{2} K_{app}$$

where, *b* ≈ *Δ*(*2πq*)^2^, and when *δ *≈* Δ* (violation of narrow pulse approximation), the effective diffusion time ‘*τ*’ (*τ* = *Δ* − *δ/3*) should be used in Eq. (5) instead of *Δ*, i.e.,

7 $$b = \gamma^{2} G^{2} \delta^{2} \tau$$

By measuring the attenuated signal with multiple b-values, it is possible to estimate the apparent diffusivity (*D*_*app*_) and apparent diffusion kurtosis (*K*_*app*_) along a specific diffusion direction by fitting Eq. (6)^44^. The PDF of the diffusion kurtosis model (Eq. 6) is a Gaussian distribution with mean (*D*_*app*_) and variance ($\frac{1}{3}D_{app}^{2} K_{app} )$. Therefore, kurtosis can be used to estimate the degree of heterogeneity of the underlying structural environment.

For b-values that are significantly small (depending upon the type of tissue) and if a voxel contains a single compartment exhibiting homogeneous T2 relaxation, then for unrestricted Gaussian diffusion, the higher terms of Eq. (4) can be neglected and Eq. (4) takes the form of a well-known mono-exponential (DTI) model:

8 $$\ln \frac{S(b)}{{S(0)}} \approx - bD_{app}$$

By fitting Eq. (8) over a range of b-values, *D*_*app*_ can be estimated. However, if the range of b-values is very small (maximum b-value is too low) then the attenuation in the signal intensity will be very low and the estimation of *D*_*app*_ will be prone to noise. On the other hand, if the range is very large (max b-value is very high), then the estimation of diffusivity will incur a systematic error due to the omission of higher terms from Eq. (4).

### Diffusion MRI data processing

The noise profile of DWI at higher b-values is non-Gaussian, therefore, to reduce the influence of noise, NLMeans filter with rician noise estimation was used for each dwi dataset^45^. The noise compensated datasets were then processed to correct for Gibbs ringing artefacts^46^ and subsequently corrected for eddy current induced distortions^47^. Since, mono-exponential derived diffusion indices are highly susceptible to diffusion gradient strength and the b-value, therefore, in this study, a Kurtosis model (Eq. 6) was used to derive all diffusion-based scalar indices^48,49^. DESIGNER which is a python/matlab based API was used for preprocessing and to derive DTI and DKI derived imaging indices (https://github.com/NYU-DiffusionMRI/DESIGNER). Imaging indices fractional anisotropy (FA), mean diffusivity (MD), axial diffusivity (AD), radial diffusivity (RD), mean kurtosis (MK) and kurtosis anisotropy (KA) were derived from the kurtosis tensor^36,37^. FA measures the degree of diffusional anisotropy and has a range of 0 and 1 as it derives from a ratio of random to highly-directional diffusion from the diffusion tensor. Higher FA values suggest higher directionality of diffusion. MD represents the diffusion rate within a voxel, with higher values representing increased diffusivity or isotropy. AD represents the rate of diffusion along the principal diffusion direction. RD represents the rate of diffusion transverse to the principal diffusion direction. MK is the average diffusion kurtosis along all directions, with higher values representing increased restriction to molecular diffusion (non-Gaussian) along these dimensions; KA estimates the variability in the kurtosis and is derived from the standard deviation of the kurtosis. KA has a range from 0 to ∞ and it captures diffusional anisotropy with the additional dimensionality that is provided by the kurtosis model.

The locust brain, as with other insects, has a complex tracheal system^50,51^. These trachea supply oxygen to the locust brain and branch into fine tracheoles with an ordered arrangement facilitating gaseous exchange^50^ but will not be in the tractography visualization as they contain air in a live animal, which will not produce a signal on MRI.

For tractography based assessment, DSIStudio^52^ was used with the following parameters: FA tracking threshold 0.15, fibre length range [0.31 50] mm, angle threshold = 45 degree, step size = 0.05 mm, tracking algorithm = Euler and interpolation method = cubic. For each dataset 5000 fibre tracts were generated and exported to TrackVis^53^ compatible format for visualization. These tractography parameters are aimed to focus only on large tracts in the final fibre reconstruction and also aim to minimize the inclusion of false tracts in the visualization, which is a known difficulty of the technique^31^. Small tracts will be excluded by the restriction in fibre length range (min = 0.18 mm, max = 50 mm). Tractography parameters would further ensure exclusion of the trachea in the visualizations in the ex-vivo locust head if saline were to enter the trachea. This would occur as the FA tracking threshold of 0.15 would restrict potential tracheal system contribution to the tractography visualization; FA >  = 0.15 would dictate that if diffusivity is isotropic or close to it; which would be the case in main tracheal tracts; it should ignore those regions during fibre reconstruction. Additionally, any small tracheal branches would also excluded via the fibre length range restriction.

For region of interest (ROI) based assessment, ROIs for antennal lobe (AL), axonal tracts (Ax-tk), central complex (CC), lamina (La), lobula complex (Lox), mushroom body (MB), medulla (ME), midbrain neuropil (MN) and retina (Rt) were manually drawn using individual T2-W and mean DWI maps. The median values of FA, MD, MK and KA were extracted for each of the ex-vivo samples using the above-mentioned binary ROIs.

## Results

**Figure 1:**
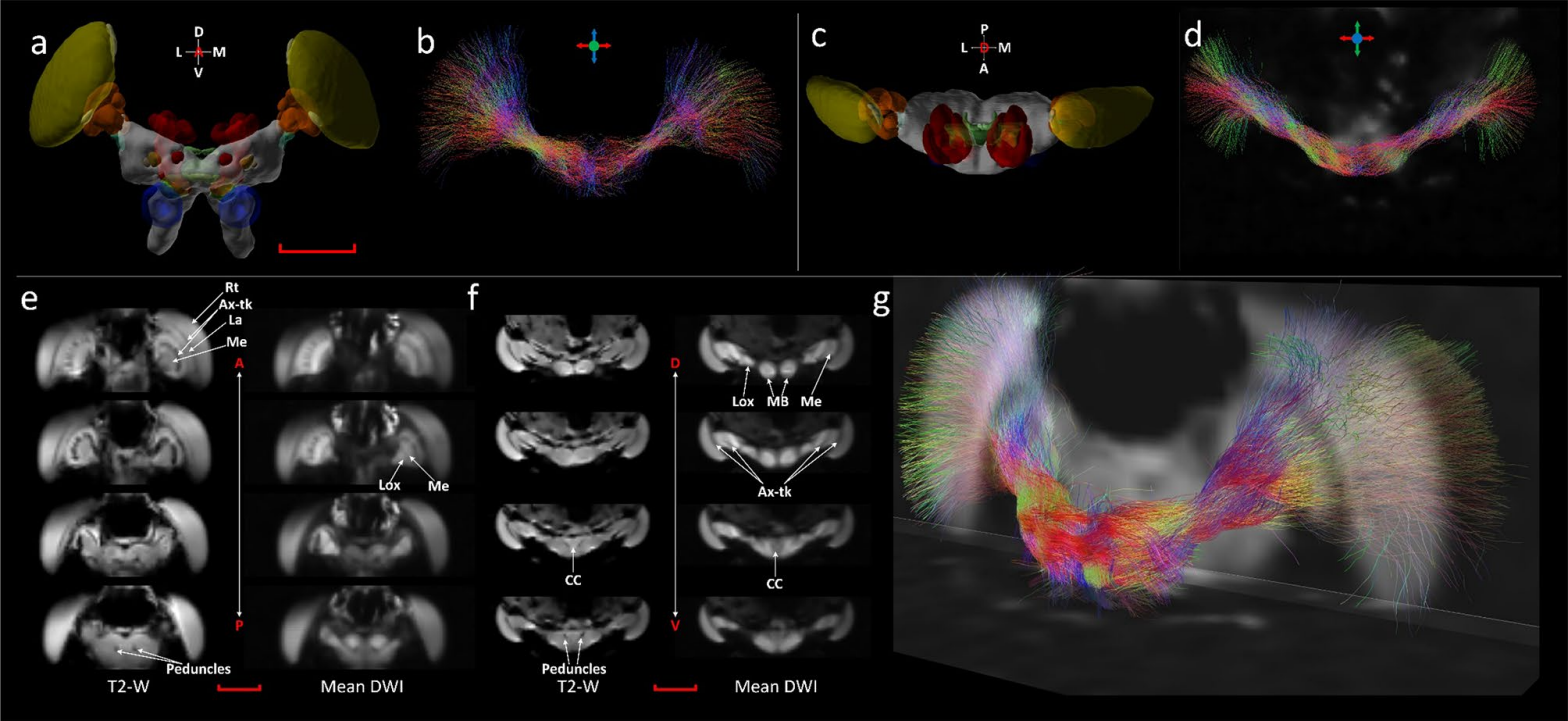
Anatomical regions and tract connectivity derived from dMRI in a live locust. (**a**) Anterior view of 3D anatomical representation taken, with permission, from http://insectbraindb.org^44^. (**b**) 3D projections of directionally encoded tracts from synaptic neuropils (coronal slice, anterior view). (**c**) Dorsal view of 3D anatomical representation taken, with permission, from http://insectbraindb.org^44^. (**d**) 3D projections of directionally encoded tracts from synaptic neuropils (axial slice dorsal view). (**e**) Coronal slice (anterior to posterior view) using T2-W and averaged DWI based contrasts. (**f**) Axial slice (dorsal to ventral view) using T2-W and averaged DWI based endogenous contrasts. (**g**) 3D view of axonal tract projections from synaptic neuropils. In (**b**) and (**d**) coloured crosshairs highlight the direction of tracts. In (**e**) and (**f**) T2-W maps, regions with longer transverse relaxation (T2-relaxation) are hyperintense and regions exhibiting shorter transverse relaxation are hypointense. In averaged DWI based maps, regions of low diffusivity are hyperintense and regions of high diffusivity are hyperintense. *Ax-tk* axonal tracts, *CC* central complex, *La* lamina, *Lox* lobula complex, *MB* mushroom body, *Me* medulla, *P* peduncles, *Rt* retina. Scale bar 500 µm.

dMRI enabled anatomical regions to be identified from the DWIs and subsequently tract connectivity in the locust brain to be extracted based on the diffusion encoded direction over a series of DWIs. Figure 1 shows multiple anatomical features and tract connectivity in the live locust. The 3D anatomical representations (Fig. 1a,c) were taken from http://insectbraindb.org^54^ to provide a better understanding on the identification and location of various anatomical regions using cross-modality comparison, though 3D surface reconstructions from confocal microscopy do not include the retina or the lamina. In Fig. 1b,d,g, the directions of fibre tracts are color encoded, whereas Fig. 1e,f identify anatomical regions using T2-W and averaged DWI based contrasts. Since we removed the antennae from the animal during preparation, the remainder of the antennal nerve was excluded from the visualization.

**Figure 2:**
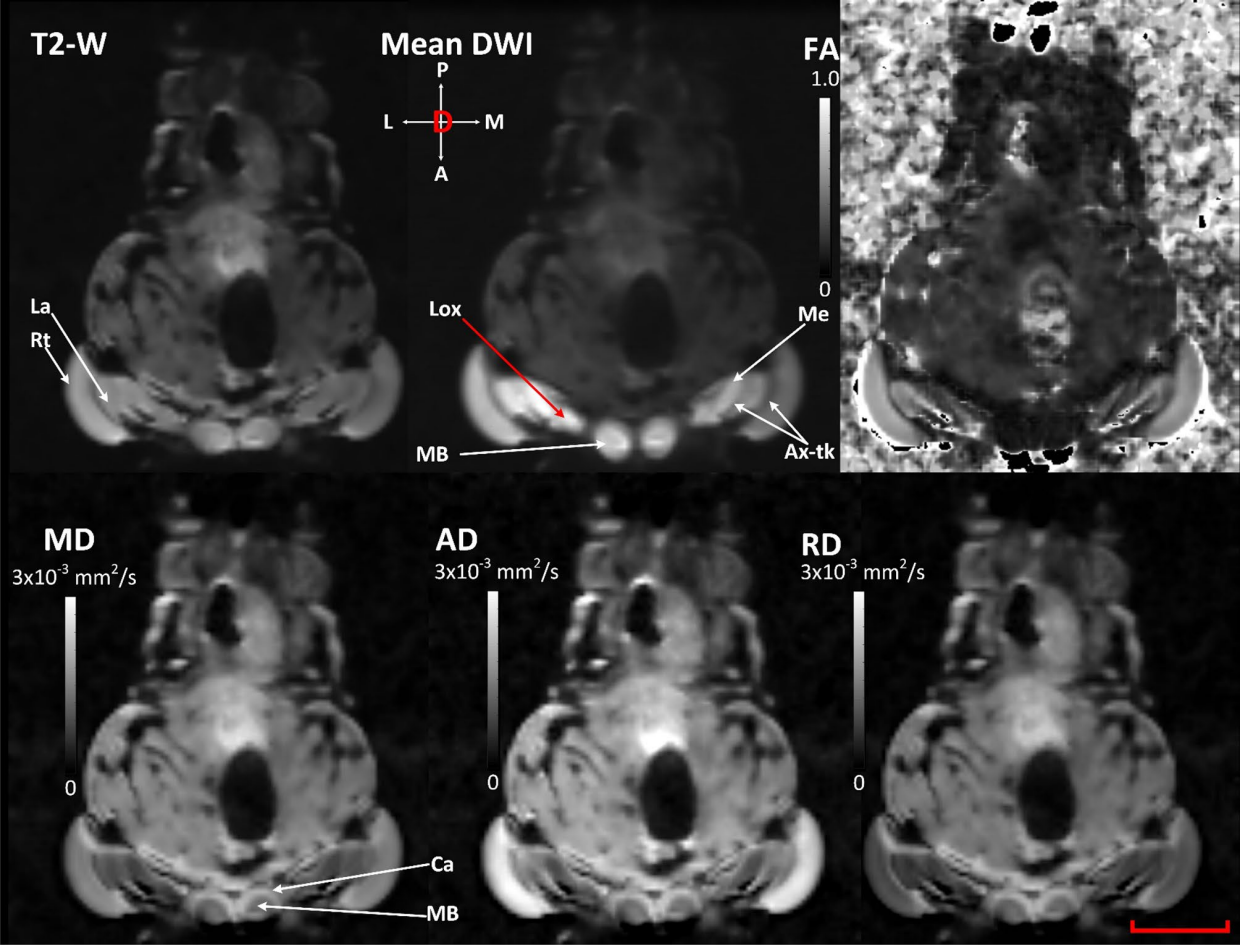
Axial slice, dorsal view from live sample illustrating various anatomical structures and their diffusivity profile using qualitative and quantitative scalar maps. T2-W and mean DWI based contrasts are qualitative in nature and their pixel/voxel intensity scales are arbitrary. FA, MD, AD and RD are quantitative scalar maps, in this study they were derived from DKI model. In T2-W map, regions exhibiting longer transverse relaxation (T2-relaxation) are hyperintense and regions exhibiting shorter transverse relaxation are hypointense. In averaged DWI based map, regions of low diffusivity are hyperintense and regions of high diffusivity are hyperintense. FA measures the degree of directional (diffusivity) anisotropy and has a range between 0 and 1. High FA values suggest higher directionality of diffusion. MD represents the mean diffusion rate within a voxel, with higher values representing increased diffusivity or isotropy. AD represents the rate of diffusion along the principal diffusion direction. RD represents the rate of diffusion transverse to the principal diffusion direction. The range of MD, AD and RD is from 0 to 3 × 10^–3^ mm^2^/s. *Ax-tk* axonal tracts, *Ca* Calyx, *La* lamina, *Lox* lobula complex, *MB* mushroom body, *Me* medulla, *P* peduncles, *Rt* retina. Scale bar 500 µm.

**Figure 3:**
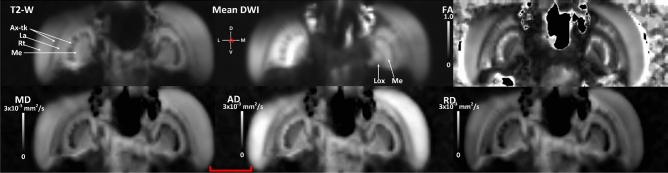
Coronal slice, anterior view from live sample illustrating anatomical structures and their diffusivity profile using qualitative and quantitative scalar maps. T2-W and mean DWI based contrasts are qualitative in nature and their pixel/voxel intensity scales are arbitrary. FA, MD, AD and RD are quantitative scalar maps, in this study, they were derived from DKI model. In T2-W map, regions exhibiting longer transverse relaxation (T2-relaxation) are hyperintense and regions exhibiting shorter transverse relaxation are hypointense. In averaged DWI based map, regions of low diffusivity are hyperintense and regions of high diffusivity are hyperintense. FA measures the degree of directional (diffusivity) anisotropy and has a range between 0 and 1. High FA values suggest higher directionality of diffusion. MD represents the mean diffusion rate within a voxel, with higher values representing increased diffusivity or isotropy. AD represents the rate of diffusion along the principal diffusion direction. RD represents the rate of diffusion transverse to the principal diffusion direction. The range of MD, AD and RD is from 0 to 3 × 10^–3^ mm^2^/s. *Ax-tk* axonal tracts, *La* lamina, *Lox* lobula complex, *Me* medulla, *Rt* retina. Scale bar 500 µm.

In addition to the anatomical features, quantitative metrics can be extracted from the DWIs. Figures 2 and 3 show axial and coronal slices, respectively, with a number of contrasts derived from the acquired DWIs and the Kurtosis model. The hyperintensities in T2-W contrast in Figs. 2 and 3 (supplementary Fig. S1) are from fat bodies and regions affected by air-tissue susceptibility induced distortions. In the mean DWI based contrasts, the regions of hinder/restricted diffusion exhibited much stronger contrast^31^.

Using this multi-contrast approach, it is possible to identify various regions, such as Retina (Rt), Lamina (La), Medulla (Me), peduncles (P), axonal tracts (ax-tk), Mushroom body (MB), Lobula complex (Lox) and antennal lobe (AL)^55^. Figures 2 and 3 (Supplementary Fig. S1) also show the fractional anisotropy (FA), mean diffusivity (MD), axial diffusivity (AD) and radial diffusivity (RD) maps. Using these quantitative maps of diffusivity, it is possible to identify several synaptic microdomains and infer axonal tract bundles. These maps provide a quantitative insight of the microstructural environment. The FA map shows high contrast in Rt, La, Me and Lox (Figs. 2 and 3). This would be indicative of these regions having a high directionality which would be consistent with the visual processing pathway, from the retina through the optic lobes towards the central brain. In contrast, the optic lobe regions are hypointense in mean, axial and radial diffusivity maps (Figs. 2 and 3), which is thought to relate to higher levels of structural complexity. This finding would reflect the layering of synaptic networks, with each neuropil known to consist of both columnar arrangements of neurons and also local interneurons^56^.

**Figure 4:**
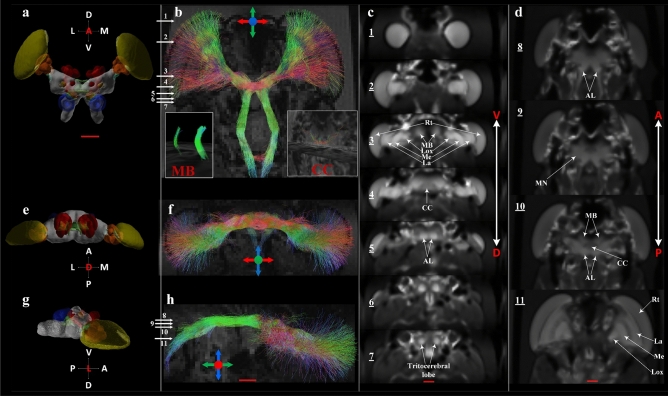
Anatomical regions and tract connectivity derived from dMRI in an ex-vivo locust. (**a**) Anterior view of 3D surface reconstruction taken from http://insectbraindb.org^44^. (**b**) Projections of directionally encoded tracts from synaptic neuropils in one of the ex-vivo samples. The colours in crosshair represent the direction of 3D tract projections (coronal slice, anterior view). (**c**) Insets 1–7 show locations of anatomical structures in axial planes (ventral to dorsal) using the map obtained from the arithmetic average of 26 diffusion-encoding directions. (**d**) Insets 8–11 show locations of anatomical regions in coronal planes (anterior to posterior) using averaged DWIs. (**e**) Dorsal view of 3D surface reconstruction taken from http://insectbraindb.org^44^. (**f**) Directionally encoded tracts from synaptic neuropils (axial slice, dorsal view). The colours in crosshair represent the direction of 3D tract projections. (**g**) Lateral view of 3D surface reconstruction taken from http://insectbraindb.org^44^. (**h**) Projections of directionally encoded tracts from synaptic neuropils (sagittal slice, lateral view). The colours in crosshair represent the direction of 3-D projections. *AL* antennal lobe, *CC* central complex, *La* lamina, *Lox* lobula complex, *MB* mushroom body, *Me* medulla, *MN* midbrain neuropil, *P* peduncles, *Rt* retina. Scale bar 500 µm.

While the focus was put on the large tract connectivity in this study and a simplified visualization, further information on the tracts is available from the images. For instance; fibres between the lamina and medulla and similarly, fibres between medulla and Lox, which are known to form chiasms are identifiable (highlighted as Ax-tk in figures). This is possible as fibre tracts have been directionally encoded in the figures with the colour of the fibre representing its orientation. From Fig. 1b, the chiasms are identifiable in the tone of blue/purple. Therefore a perfectly vertical fibre would be displayed in pure blue colour (as marked by the crosshair). Similarly, perfectly horizontal fibres would be displayed in pure red. Any change in the orientation of the fibre from three orthogonal directions are represented by the mixture of multiple colours. From Figs. 1e,f, 2, 3 and 4c,d, the regions of darker shades between adjacent lamina and medulla and between Lox and medulla clearly highlight those chiasms (Ax-tk). The chiasms can also be identified in the ex-vivo locust in the fractional anisotropy (FA) modulated directionally encoded colour (DEC) map (supplementary Fig. S1b, Ax-tx, highlighted by yellow arrows).

Using the color encoded projections of inferred axonal tracts from synaptic neuropils, it is possible to estimate the micro-diffusion environment (Figs. 1 and 4). The bundles of axonal fibres connecting the synaptic centers of the optic lobes towards the brain can be seen from Figs. 1b,d,g and 4b,f,h. Regions that show high contrast in Fig. 4c (insets 1–7) are Rt, MB, Lox, Me, La, CC, AL and Tritocerebral lobe. Similarly, Fig. 4d (insets 8–11) show good classification of midbrain neuropil (MN), MB, AL, La, Me and Lox. The central complex (CC), which has high synaptic density appears hypointense in the averaged DWI map, whereas, T2-W contrast shows clear delineation of CC and hypo-intense peduncles (P) (Figs. 1 and 4). The insets in Fig. 4b show the 3-dimensional representation of tracts in MB and CC using manually delineated region-specific (ROI-based) fibre tractography. Hence, use of dMRI at micro-scale with multi-contrast maps can be used to identify synapse-rich neuropil sub-structures and infer axonal tract bundles.

Rotationally invariant measures such as FA and MD and rotationally variant measures such as RD and AD can be used to extract quantitative information from various micro-structures as can be visualized from Figs. 2 and 3 for live locust and supplementary Figs. S2 and S3 for the ex-vivo samples. From three ex-vivo samples, the median value of rotationally invariant measures (FA, MD, MK and KA) were extracted using manually delineated region of interest based binary maps of AL, CC, Rt, Me, MB, La, MN, Lox and Ax-tk. From Table 1, it can be seen that Rt, La and axonal tracts showed the highest FA values, whereas, CC showed lowest median FA. Using MK and KA, the complex organization of the underlying microstructure can be quantified. For example, Lox exhibited highest kurtosis values due to its complex micro-structural (diffusion) environment mapped at 78 µm^3^ scale (Table 1), whereas, CC, despite having high synaptic density showed lower kurtosis values as well as low fibre density (Fig. 4b-inset), which indicates that much higher resolution is required to better classify high synaptic regions. Therefore the quantitative metrics from DWIs can also provide insights into the limits of the imaging technique in the locust brain. Interestingly the quantitative metrics for each region were within a similar range for each animal. In future studies for these values to have utility as baseline dMRI metrics in a longitudinal study there would need to be an increase in sample size, but the initial results are supportive that dMRI metrics could be used to examine structural differences in relation to differing physiology.

**Table 1:** Median values of Kurtosis tensor derived directionally invariant indices from selected ROIs.

	AL	CC	Rt	Me	MB	La	MN	Lox	Ax_tk
**Locust 1**									
FA	0.36	0.23	0.67	0.49	0.36	0.56	0.32	0.44	0.62
MD (mm^2^ s^−1^)	0.46 × 10^–03^	0.76 × 10^–03^	0.64 × 10^–03^	0.35 × 10^–03^	0.47 × 10^–03^	0.52 × 10^–03^	0.47 × 10^–03^	0.37 × 10^–03^	0.46 × 10^–03^
MK	1.88	0.73	1.04	2.64	2.39	1.60	1.69	2.65	2.46
KA	0.95	0.32	0.89	1.64	1.57	0.81	0.71	1.07	1.86
**Locust 2**									
FA	0.50	0.31	0.68	0.49	0.50	0.67	0.51	0.62	0.67
MD (mm^2^ s^−1^)	0.43 × 10^–03^	0.62 × 10^–03^	0.65 × 10^–03^	0.37 × 10^–03^	0.36 × 10^–03^	0.45 × 10^–03^	0.48 × 10^–03^	0.26 × 10^–03^	0.52 × 10^–03^
MK	2.23	0.75	0.90	2.51	3.52	2.03	2.02	4.60	2.48
KA	0.92	0.39	0.59	1.11	2.08	1.19	0.93	3.83	1.60
**Locust 3**									
FA	0.39	0.28	0.65	0.52	0.24	0.71	0.28	0.61	0.69
MD (mm^2^ s^−1^)	0.59 × 10^–03^	0.74 × 10^–03^	0.57 × 10^–03^	0.31 × 10^–03^	0.53 × 10^–03^	0.41 × 10^–03^	0.51 × 10^–03^	0.28 × 10^–3^	0.42 × 10^–03^
MK	2.24	0.85	0.89	2.04	1.94	1.84	1.77	4.91	2.65
KA	0.48	0.36	0.65	1.04	0.41	1.55	0.45	4.54	2.22

*AL* antennal lobe, *CC* central complex, *Rt* retina, *Me* medulla, *MB* mushroom body, *La* lamina, *MN* midbrain neuropil, *Lox *lobula complex,  *Ax-tk* axonal tracts.

## Discussion and conclusion

There is increasing evidence to support the early observations by Ramon y Cajal which suggested that there were common design principles behind the formation of neuronal networks to subserve complex brain function across phyla^1,3^. Therefore, the insect brain offers a simpler and more accessible nervous system that can add to understanding of the general principles behind how neuronal networks result in adaptable behavior^6,57^. Further the use of insect brains as a model have been proposed to enable better understanding from the molecular level for how ageing^58^, disease^59^ or environmental change^60^ impacts on both neuronal networks and resulting behavioral output.

This proof of concept study demonstrates a novel methodology for imaging insect brains. Whilst the resolution of MRI is significantly lower than that can be achieved through electron microscopy, brain regions that have been previously identified by microscopy techniques could be identified in this study^55^ and therefore dMRI can provide qualitative insights into the locust brain, for instance brain volume comparisons. Further, tractography enabled connectivity of axonal tract bundles from the optic lobe to the central brain to be inferred. However dMRI does not yet provide high enough resolution for visualization of tract connectivity in the central brain of the locust nor can it reliably detect smaller tracts, and certainly cannot achieve the synaptic level resolution of electron microscopy. Significantly though the methodology enables standard dMRI metrics to be extracted that can enable a quantitative insight into the microstructural environment of larger axonal tracts in the locust’s optic lobes as viewed in both live and ex-vivo intact locust heads. The metrics can detect any underlying alterations in tissue that cannot be seen via visualization. These diffusion metrics could be used to quantify longitudinal structural changes in the insect brain that occur due to environmental stressors or ageing. The development of the locust as a first model animal for DWI in an insect is appropriate since locusts are known to exhibit an extreme form of neural plasticity driven by environmental factors with substantial differences between the brains of the two phases of locust^22^.

Previously a number of studies have tried to use MRI to image the internal organs of various small animals, including insects, but with limited success^61–63^. These studies mostly employed anatomical (qualitative) scans to observe features such as exoskeleton, guts, ovaries and muscles. Manganese enhanced magnetic resonance imaging (MEMRI); where intracellular accumulation of manganese is used to infer neuronal activity; has been applied to the locust to monitor neuronal activity concurrent with locomotion^64^. MEMRI can also be applied to trace identifiable neuronal circuits, and this has been successfully applied to *Aplysia*^65^ but not yet to insects. To the best of our knowledge, there is only one study to date which tried to classify internal structures of insects, (*Drosophila melanogaster*) using FLASH sequence and DWI based contrast^35^. However, that study only applied diffusion sensitizing gradient along the slice selection gradient. Due to a single diffusion encoding direction and lack of quantitative assessment (apparent diffusion coefficient along slice select direction ADC_Z_), it is difficult in that study to comprehend a more comprehensive diffusion profile of the underlying microstructures. This may be the reason that the authors of that study could not classify major axonal tract bundles and regions containing cell bodies. In this study we applied diffusion sensitizing gradients in 26 diffusion encoding directions and used 4 b-values (3 shells). To classify various regions, we used number of contrasts and by employing a Kurtosis tensor we were able to classify the diffusion patterns in neuropils (small axons, dendrites and synaptic terminals) as well as in regions where there are cell bodies, which are usually difficult to assess using DWI or simple diffusion models. However, due to the limitations of resolution it may still be difficult to apply dMRI to visualize axonal tracts in smaller insect brains such as *Drosophila* even with the additional diffusion encoding directions.

The application of the dMRI to visualize the insect brain and optic lobes could be particularly beneficial for understanding the formation of neuronal circuits and the potential impact of physiological or environmental change. Although this study demonstrated that the application of dMRI to understand the central brain may be limited at this time by lack of resolution of the technique, the micro-scale diffusion environments of the optic lobes could be reliably quantified in our proof-of-concept study, with consistency in diffusion metrics extracted from the ex-vivo head of different animals. Future studies should be able to implement the methodology to quantify longitudinal structural changes in the nervous system of the locust. In the insect, the visual system is proportionally by far the largest region as may be expected by the necessity of vision for the animal and the demanding computation it requires. There has been extensive prior characterization of the visual system of insects, particularly in flies but also in locusts and bees from the first observation of the similarity in the organization for insect and mammalian visual systems^56^. The insect visual system is known to be energetically demanding on the animal, with the circuitry highly evolved to efficiently and rapidly transmit information to the central brain^66^. Interestingly there is evidence that there is experience dependent plasticity in the fly optic lobe^67^. Although the visual system in the insect is already well characterized through electron microscopy and also electrophysiology, the application of dMRI has the potential to detail dynamic changes that occur in visual system wiring that is necessitated by changing demands on the animal. Interestingly, in the locust there is also known plasticity in the optic lobe with differences in the relative proportions of neuropils within the optic lobes between solitarious and gregarious locusts^22^, with these changes ascribed to multifactorial environmental and dietary changes. dMRI could enable these changes to be monitored longitudinally and help to provide insights into the key determinants of the neural plasticity.

An important addition dMRI provides to the imaging of the insect brain is the availability of dMRI derived quantitative indices (Figs. 2 and 3) as well as fibre tractography (Figs. 1 and 4). DTI metrics including FA have already demonstrated value in human studies of patients with brain trauma or neurodegeneration^30^. dMRI derived metrics have the potential to quantify changes in the organization of the neuronal microstructure that occurs due to environmental stresses or aging. DTI and DKI are advantageous techniques for characterizing axonal changes as there may not be overt axonal damage even in the presence of significant functional damage^68^. Therefore better insights into physiological changes in the brain are likely to be gained from this method than through the use of electron microscopy technique, even though electron microscopy has far better spatial resolution. For instance in this study even though the voxel size is 78µm^3^, by acquiring DWIs in multiple diffusion directions each voxel contains information related to underlying structures that are much smaller than the spatial resolution enabling the microstructural diffusion environment to be quantified. There is evidence that measures derived from DWI via DTI are sensitive enough to detect pathological changes in axonal architecture associated with osmotic swelling that occurs as a consequence of alterations in membrane polarization^17^. Such axonal changes are thought to be indicative of Wallerian degeneration or axonal self-destruction that occurs in disease and after trauma^68^. Understanding the mechanisms behind the altered architecture of axons is important as this pathological process is thought to be characteristic of many neurodegenerative diseases. Although there are morphological differences between insects and vertebrates, similarities at the cellular and molecular level; with both neural signaling and the innate immune response highly conserved from insects through to mammals^69^; have led to insects being proposed as important models for understanding the biological pathways underlying axonal self-destruction. With the availability of dMRI in an insect model system there is the potential for a methodology that could be used to study axonal damage from the molecular^59^ through to the systems level.

The benefits of micro-dMRI being applied to an insect model may be twofold. Whilst the widely used dMRI derived metrics like FA, KA, MK and MD have been found to have prognostic value in human patients the exact alterations that they are capturing are not completely understood^70,71^. Non-mammalian models have previously been found to be useful in aiding understanding of the biological basis of DWI metrics^72–74^. MR imaging of in vivo and ex vivo nerves across many different species has been used to explore the biological basis of diffusion parameters, and the linkage of these parameters to alterations in underlying tissue structure^73^. It may be that these metrics are particularly sensitive to a given cellular change, for instance changes in glia. Although there are morphological differences, the insect brain contains glia with analogous functional types to those observed in mammalian brains^75–77^. Whilst alterations in glia are the most frequently proposed reason for changes in dMRI metrics, axon diameter, packing density and membrane permeability should also be considered^72,73^. It could be easier in an insect model to determine the mechanistic basis for micro-dMRI derived metrics that may provide valuable understanding when dMRI is applied in other animals, including humans.

The novel application of dMRI and its signal representation schemes-based methodology for insect brain imaging is preferable over other possible imaging techniques for many reasons. Non-invasive imaging techniques such as MRI do not interfere with the organization of underlying microstructure, as opposed to histology and microscopic based imaging techniques^35^. Therefore such techniques are better suited for non-destructive biological imaging in live animals and so open up new possible research avenues; the technique could be applied to characterize neuronal connectivity over an animal’s lifespan. Additionally, despite the development of processing pipelines for the reconstruction of electron microscopy data^10^ this technique currently remains time consuming, with the limitation increasing with the size of the neuronal network^6^. However, at this time there remain some limitations in the dMRI technique and therefore the methodology is currently not a replacement for traditional electron microscopy methods but rather provides supplementary imaging that provides additional value for the use of insects in neuroscience. For instance, despite the use of multiple diffusion gradients in DKI providing an improvement on the DTI in terms of inferring the underlying biological structure, there is still the possibility for error and tracts can be erroneously inferred or missed^31,78^. However, in future, the use of electron microscopy data in combination with MRI may help to reduce errors in tractography or missed tracts. Technical improvements in MRI can reduce the chance of errors in the extraction of tracts, for instance higher dimensionality of the diffusion model or higher field MR. Within larger invertebrate brains, such as the squid, dMRI has been able to both confirm the presence of connectivity previously established by microscopy techniques, but also identify many previously undescribed pathways^79^.

dMRI will enable a different and yet complementary approach to existing imaging methodologies. The application of micro MRI and dMRI modelling to the insect brain may help to provide an increase understanding of the biological underpinnings of these widely used metrics as well as understand and characterize dynamic changes in neural circuits in insect brains. The linkage of metrics from dMRI and DKI with existing well established techniques to characterize functional output of the neuronal network could provide a powerful methodology to unravel how neuronal structure impacts on behavioural output.

## Supplementary Information

Supplementary Figure 1.

Supplementary Figure 2.

Supplementary Figure 3.

Supplementary Figure Legend.

## Data Availability

The raw dMRI volume data used in the study are available from the corresponding author on reasonable request.
