# Supplementary figures and images for: Elucidating the complex organization of neural micro-domains in the locust Schistocerca gregaria using dMRI
Source: Sci Rep. 2021 Feb 9;11:3418. doi: 10.1038/s41598-021-82187-3 (PMC7873062; doi:10.1038/s41598-021-82187-3)

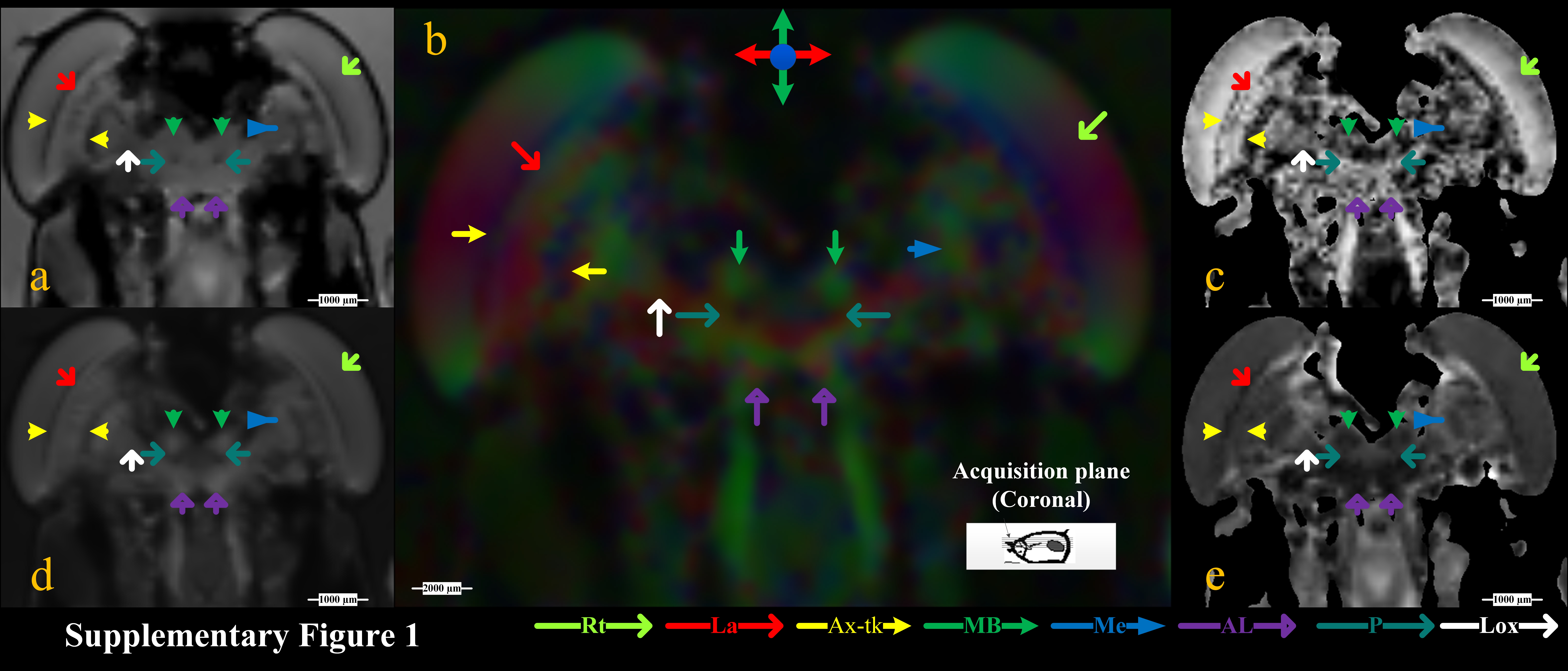

Supplement: Supplementary file 1 — Supplementary Figure 1. [file 41598_2021_82187_MOESM1_ESM.jpg]

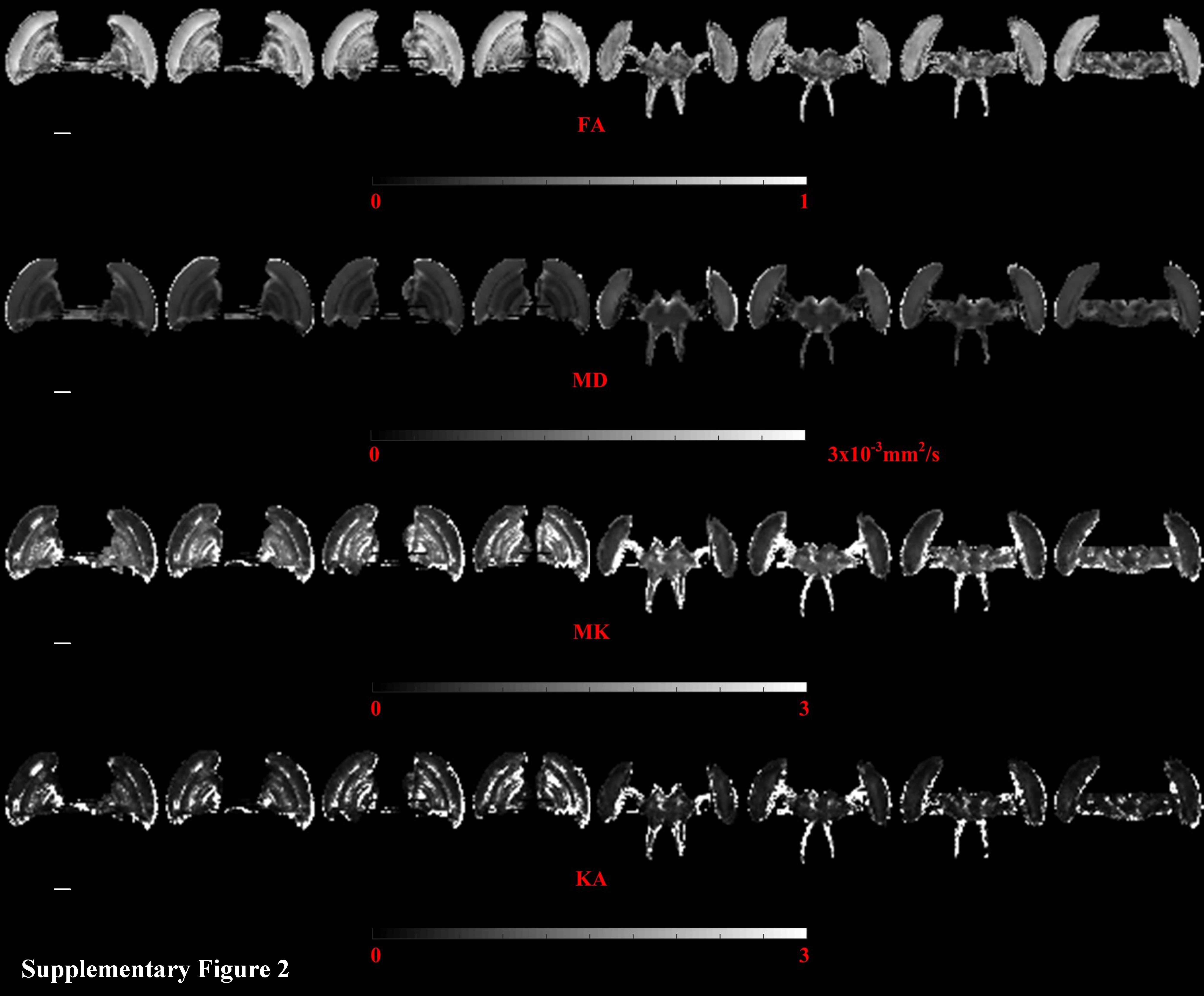

Supplement: Supplementary file 2 — Supplementary Figure 2. [file 41598_2021_82187_MOESM2_ESM.jpg]

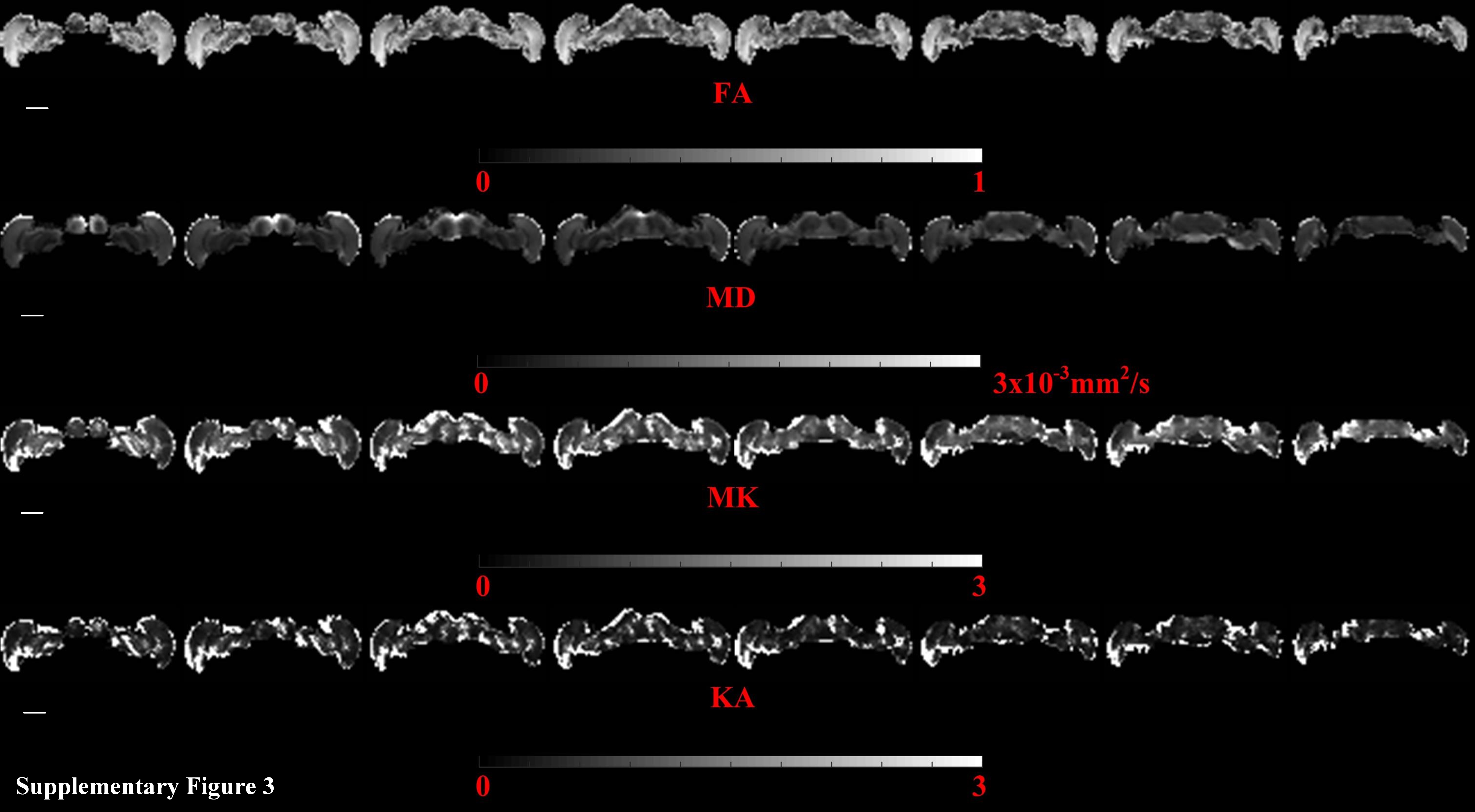

Supplement: Supplementary file 3 — Supplementary Figure 3. [file 41598_2021_82187_MOESM3_ESM.jpg]
