# Supplementary material for: Elucidating the complex organization of neural micro-domains in the locust Schistocerca gregaria using dMRI
Source: Sci Rep. 2021 Feb 9;11:3418. doi: 10.1038/s41598-021-82187-3 (PMC7873062; doi:10.1038/s41598-021-82187-3)
Supplement: Supplementary file 4 — Supplementary Figure Legend. [file 41598_2021_82187_MOESM4_ESM.docx]

**Supplementary Figure Legends**

**Supplementary Figure 1:** Coronal slice, anterior view from ex-vivo sample no.2 illustrating various anatomical structures and their respective diffusion profiles. (a) b0 or T2-W image; regions with longer transverse relaxation (T2-relaxation) are hyperintense, whereas, regions exhibiting shorter transverse relaxation are hypointense, for example, cornea has the shortest T2-relaxation and La has comparatively longer T2-relaxation time, (b) Fractional anisotropy (FA) modulated directionally encoded color (DEC) map, which can be used as a visual representation of the orientation of the principal diffusion direction. (c) FA map highlighting regions of low and high anisotropy. The range of FA is from 0 (isotropic diffusion) to 1 (highly restricted diffusion) (d) map obtained from the arithmetic average of 26 diffusion-encoding directions, regions of low diffusivity are hyper-intense and regions of high diffusivity are hypo-intense. (e) Mean diffusivity (MD) map, regions of low diffusivity are hypo-intense whereas, regions of high diffusivity are hyper-intense. The range of MD is from 0 to 3x10^-3^ mm^2^/s. Rt: retina; La: lamina; Ax-tk: axonal tracts; MB: mushroom body; Me: medulla; AL: antennal lobe; P: peduncles; Lox: lobula complex.

**Supplementary Figure 2**: Kurtosis tensor derived directionally invariant indices. Coronal plane (ex-vivo sample no. 1). The indices (FA, MD, MK, KA) were only calculated in a manually drawn mask which roughly covers the region of interests. Scale bar 500 µm.

**Supplementary Figure 3**: Kurtosis tensor derived directionally invariant indices. Axial plane (ex-vivo sample no. 1). The indices (FA, MD,MK,KA) were only calculated in a manually drawn mask which roughly covers the region of interests. Scale bar 500 µm.
